# Immune Thrombocytopenia in an Adult With X‐linked Agammaglobulinemia: A Case Report

**DOI:** 10.1002/jha2.1101

**Published:** 2025-04-24

**Authors:** Takeaki Matsunaga, Ken Naganuma, Noriko Tanabe, Yoshiko Mori, Marino Nagata, Shuji Momose, Yasushi Kubota

**Affiliations:** ^1^ Department of Hematology, Saitama Medical Center Saitama Medical University Saitama Japan; ^2^ Department of Clinical Genetics, Saitama Medical Center Saitama Medical University Saitama Japan; ^3^ Department of Pathology, Saitama Medical Center Saitama Medical University Saitama Japan; ^4^ Department of Transfusion Medicine and Cell Therapy, Saitama Medical Center Saitama Medical University Saitama Japan; ^5^ Department of Clinical Laboratory Medicine Saga‐Ken Medical Centre Koseikan Saga Japan

**Keywords:** Bruton tyrosine kinase, hypogammaglobulinemia, immune thrombocytopenia, platelet, X‐linked agammaglobulinemia

## Abstract

In patients with X‐linked agammaglobulinemia (XLA), serum immunoglobulins are almost completely lacking. The prevalence of autoimmune diseases is low in XLA compared with other primary immunodeficiency diseases because antibodies are absent in XLA. Immune thrombocytopenia (ITP) is considered an antibody‐mediated disease characterized by increased platelet destruction, and adult‐onset ITP in XLA has not been reported in detail. The case of a 29‐year‐old Japanese man with XLA and ITP is described. The patient was treated with prednisolone and intravenous immunoglobulins, resulting in rapid improvement of thrombocytopenia. Clinicians should consider co‐existing ITP when progressive thrombocytopenia is observed in a patient with XLA.

## 1 Introduction

X‐linked agammaglobulinemia (XLA) is a congenital immunodeficiency disorder that results from impaired B‐cell differentiation due to Bruton tyrosine kinase (BTK) mutations, leading to a decrease in mature B‐cells, impaired antibody production, and hypogammaglobulinemia [1, 2, 3]. Thus, complications of autoimmune diseases with antibody‐mediated mechanisms are less common in XLA than in other primary immunodeficiency diseases. Immune thrombocytopenia (ITP) is an autoimmune disorder characterized by increased platelet destruction and impaired platelet production from bone marrow megakaryocytes. Although the cause of ITP is not entirely understood, it has been believed that ITP is mediated by autoantibodies [4]. A study reported that 2% of XLA patients had thrombocytopenia, but all were pediatric patients and it likely occurred secondary to infection [5]. Furthermore, there are very few detailed case reports of the diagnosis and treatment of ITP in XLA patients. A case of ITP in an adult case of XLA is described.

## 2 Case Presentation

When the patient was a 3‐year‐old boy, he developed pyothorax, which led to his diagnosis of agammaglobulinemia. He was started on routine immunoglobulin replacement therapy (IgRT). After the beginning of IgRT, no severe infection was observed.

**FIGURE 1:**
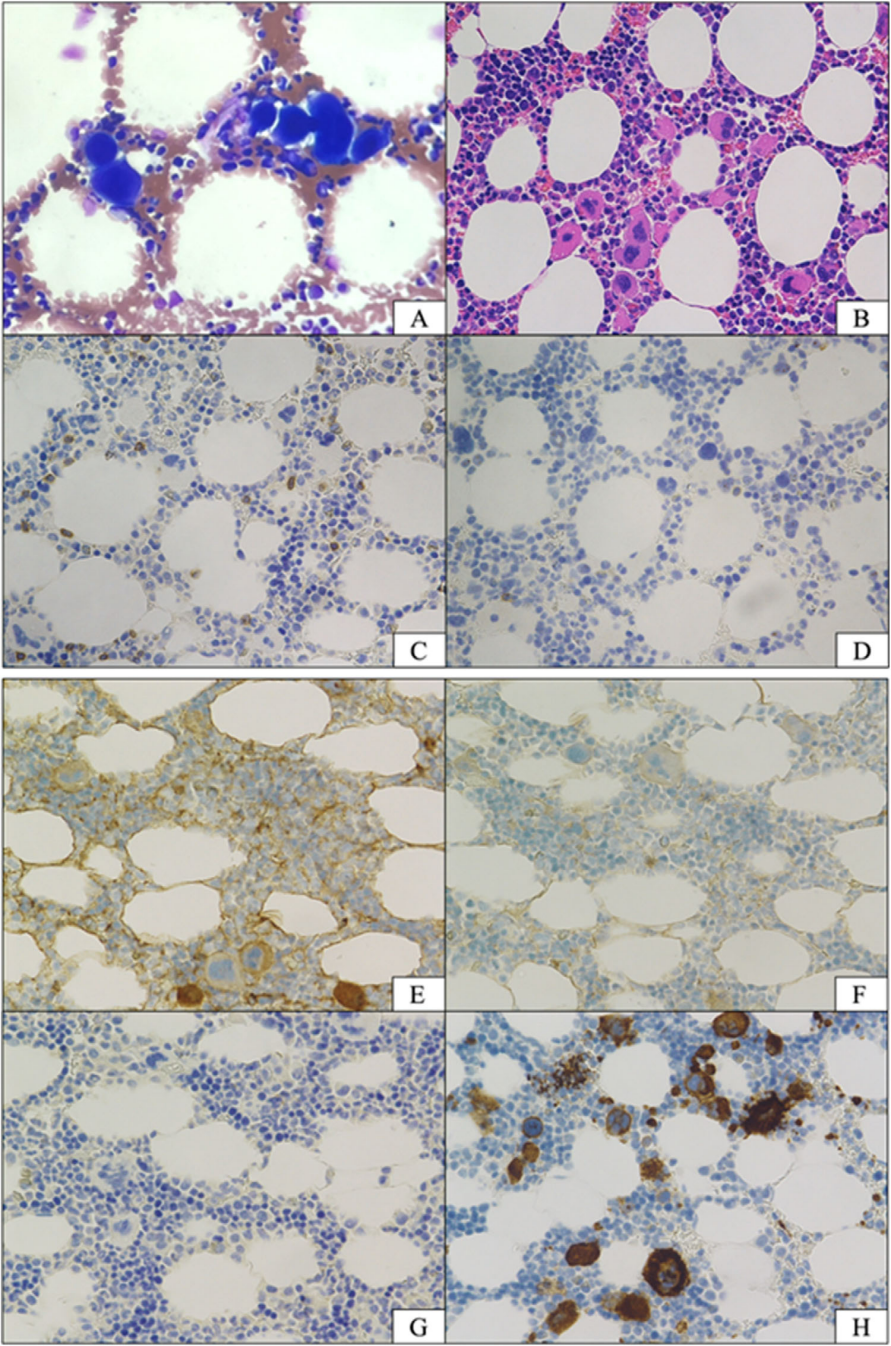
Bone marrow aspiration smears and clot section of the patient. (A) Aspiration smears showing increased megakaryocytes. (May‐Giemsa stain; original magnification ×1000) Megakaryocyte counts were increased to 237/µL (reference, 50–150/µL). (B) Clot section showing markedly decreased B cell lineage and increased immature megakaryocytes. (hematoxylin‐eosin stain; original magnification ×400) (C–H) Immunostaining of bone marrow cells. (C) CD3, (D) CD20, (E) κ, (F) λ, (G) CD138, and (H) CD41.

At the age of 29 years, the patient was referred to our department for thrombocytopenia that had progressed over a 2‐month period. Privigen (pH4‐treated human normal immunoglobulin; CSL Behring, King of Prussia, PA, USA) had recently been routinely administered. There were no findings suggestive of infection in the months prior to the onset of thrombocytopenia. At his first visit, laboratory tests showed a platelet count of 11×10^9^/L. The patient's laboratory test results are summarized in Table 1. The immature platelet fraction was 7.0% (reference range, 1.1%–6.1%). Coagulation tests were normal. Liver function tests showed slightly increased hepatobiliary enzymes, a condition that had been previously recognized. IgG, IgA, and IgM levels were 2.87 (reference, 8.61–17.47) g/L, less than 0.05 (reference, 0.93–3.93) g/L, and less than 0.02 (reference, 0.33–1.83) g/L, respectively. Tests for anti‐platelet antibodies were negative, and platelet‐associated IgG levels were normal at 32 (reference, 0–46) ng/10^7^ cells. Laboratory tests showed no findings suggestive of *Helicobacter pylori*, Epstein‐Barr virus, or cytomegalovirus infection. Vital signs and physical examination were unremarkable, and whole‐body computed tomography showed no evidence of other infections. There was also no swelling of the lymph nodes that would suggest malignant lymphoma. Bone marrow aspiration showed normocellular marrow with an increased number of immature megakaryocytes (Figure 1A,B,H) and there were no findings of dysplasia or lymphoma cell invasion. The karyotype of the bone marrow cells was 46, XY. These findings led to the clinical diagnosis of ITP. Neither CD20‐positive nor CD138‐positive cells were observed (Figure 1D,G). Flow cytometry analysis of peripheral blood (PB) showed that CD19‐positive B cells accounted for less than 1%. Both CD4‐positive and CD8‐positive cell counts were normal. Plasma thrombopoietin levels were not examined.

**TABLE 1:** Laboratory test results of the patient's peripheral blood.

Component	Value	Reference range
White blood cell count, cells×10^9^/L	9.9	3–9.4
Neutrophils, %	77	38.5–80.5
Eosinophils, %	1	0–8.5
Monocytes, %	8	2–10
Lymphocytes, %	14	16.5–49.5
Hemoglobin, g/L	138	137–168
Mean corpuscular hemoglobin concentration, fL	82.9	84.4–101.4
Platelet count, cells×10^9^/L	30	158–348
Immature platelet fraction, %	7.0	1.1–6.1
Total protein, g/L	55	66–81
Albumin, g/L	36	41–51
Immunoglobulin G, g/L	2.87	8.61–17.47
Immunoglobulin A, g/L	less than 0.05	0.93–3.93
Immunoglobulin M, g/L	less than 0.02	0.33–1.83
Serum creatinine, µmol/L	70.72	57.46–94.59
Blood urea nitorogen, mmol/L	5.0	2.86–7.14
Aspartate aminotransferase, µkat/L	1.14	0.22–0.50
Alanine aminotransferase, µkat/L	1.32	0.17–0.70
Total bilirubin, µmol/L	6.84	6.84–25.66
Lactate dehydrogenase, µkat/L	3.27	2.07–3.71
Prothrombin time ratio	1.02	0.8–1.2
Activated partial prothrombin time, seconds	32.8	24.1–31.7
D‐dimer, nmol/L	2.46	0.00–5.42
Anti‐nuclear antibody, titer	less than 1:40	less than 1:40
Double‐stranded DNA IgG antibodies, IU/mL	less than 2	0–12
Anti‐ribonucleoprotein antibody, U/mL	less than 2	0–9
Anti‐Sjögren's syndrome‐related antigen A, U/mL	less than 1	0–9
Anti‐cardiolipin IgG antibodies, U/mL	less than 2.6	0–20
Anti‐cardiolipin IgM antibodies, U/mL	less than 1.0	0–20
Anti‐β_2_‐glycoprotein 1 IgG antibodies, U/mL	less than 6.4	0–20
Anti‐β_2_‐glycoprotein 1 IgM antibodies, U/mL	less than 1.1	0–20

**FIGURE 2:**
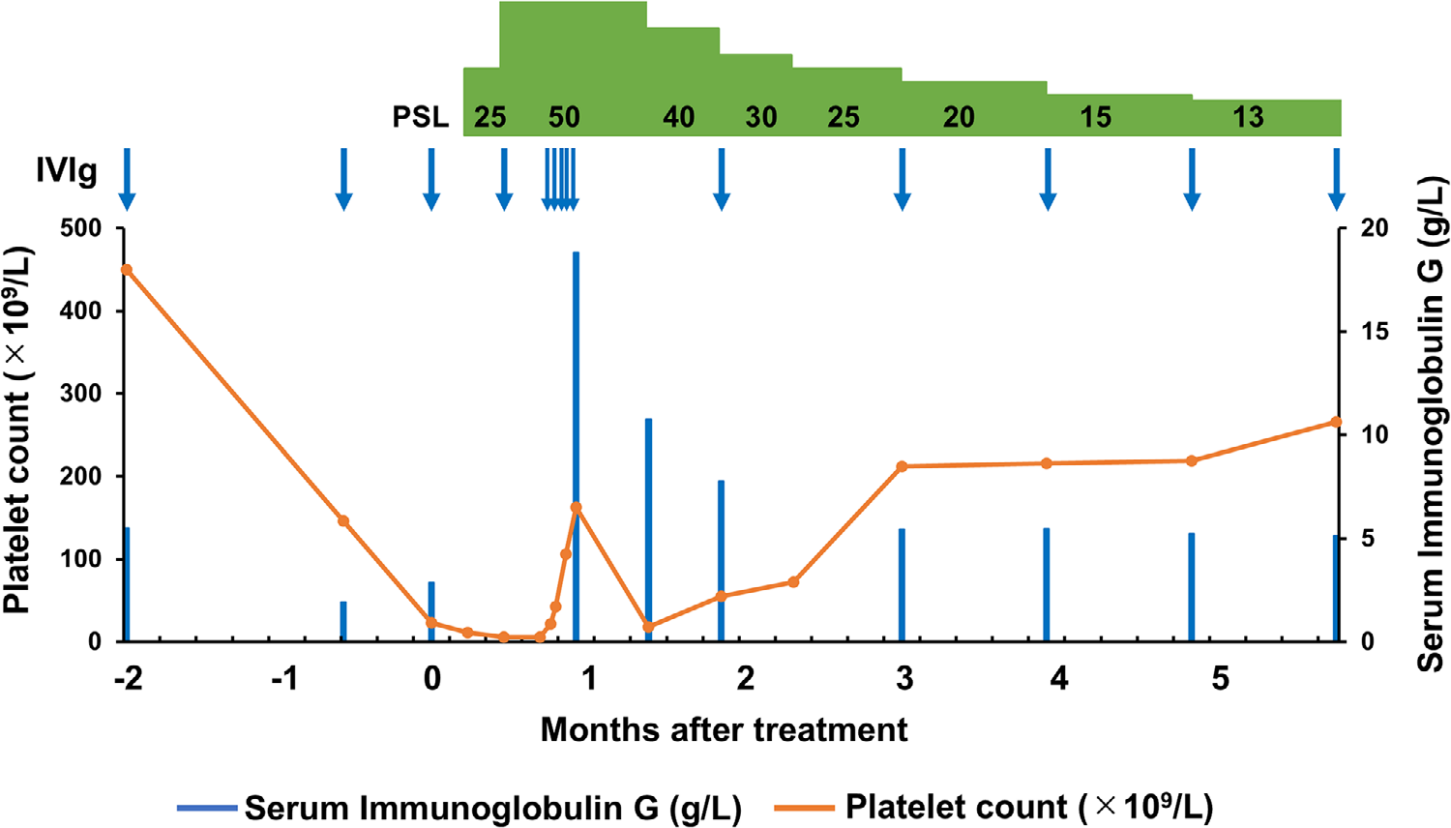
Patient's clinical course. Platelet counts and immunoglobulin G levels throughout the clinical course are shown. PSL: prednisolone, IVIg: intravenous immunoglobulin therapy.

Prednisolone (PSL) 25 mg/day (0.5 mg/kg/day) was started, but no increase in the platelet count was observed. The PSL dosage was increased to 50 mg/day. On day 14 from starting PSL, a submucosal hematoma of the oral cavity persisted, and a five‐day course of intravenous immunoglobulin (IVIg) with Venilon‐I (freeze‐dried sulfonated human normal immunoglobulin; Teijin Pharma, Tokyo, Japan, 0.4 g/kg/day) was administered. The platelet counts then recovered immediately, and symptoms of bleeding disappeared. The PSL was gradually decreased, and routine IgRT was resumed with Privigen as before the onset of ITP, with no recurrence of thrombocytopenia (Figure 2).

A molecular study was performed to clarify the genetic background of agammaglobulinemia. The diagnosis of XLA was confirmed by identifying a hemizygous pathogenic variant in the germline BTK gene (NM_000061.3), specifically a change from cytosine to thymine at position 763 (c.763C > T), resulting in a premature stop codon at arginine 255 (p.Arg255Ter).

## 3 Discussion

A case of ITP in an adult patient with XLA was presented. Several cases of thrombocytopenia have been reported in pediatric patients with XLA, occurring most likely secondary to infection [5]. The occurrence of immune thrombocytopenia in adult XLA patients without signs of infection, as in the present case, seems extremely rare. It is not clear why autoimmune diseases such as ITP develop in agammaglobulinemia.

In addition to platelet clearance mediated by anti‐platelet autoantibodies, platelet destruction by cytotoxic T cells is thought to occur in ITP [6]. Patients with ITP also have decreased and dysfunctional regulatory T cells [7]. The B cell‐T cell bidirectional interaction is critical for immune homeostasis; however, the absence of B cells in circulation and lymphoid tissues in XLA patients may affect the composition and function of T cells [8]. Several studies have shown that patients with XLA have T‐cell abnormalities, including a decreased number of regulatory T cells [9]. Taken together, these T‐cell abnormalities may have contributed to the development of ITP, although they were not studied in detail in the present patient. The patient's platelet counts improved following treatment with IVIg and PSL; this suggests a link between autoimmunity in XLA and various factors, including anti‐platelet antibodies, cytotoxic T cells, and regulatory T cells. Further studies with a larger number of cases are required to clarify the association between XLA and ITP.

Though CD19‐positive B cells of XLA patients are typically less than 1% of PB cells, some patients have more B cells. This is called the “leaky phenomenon”, which indicates that normal BTK transcripts exist in some of the BTK‐mutated B cells and suggests that a few leaky B cells in the bone marrow can migrate into the PB [10, 11]. A previous report mentioned the relationship between leaky XLA and ITP [12]; however, in the present case, CD19‐positive B cells were found to be less than 1% of PB cells and not of leaky type XLA.

IVIg‐induced acute thrombocytopenia has rarely been reported [13]. Rapid formation of IgG‐platelet complex in the circulating blood is associated with the rapid onset and progression of IVIg‐induced thrombocytopenia [14]. However, in the present case, thrombocytopenia progressed slowly over two months. Although this patient continued to use Privigen after the onset of ITP as before, thrombocytopenia has not relapsed. Therefore, the diagnosis of ITP is being more strongly considered.

In the treatment of ITP that merges with XLA, there is still no established method. In the present case, the standard ITP treatment strategy was applied, though it is important to proceed with caution when using long‐term PSL in XLA patients due to concerns about excessive immunosuppression. In one case of steroid‐dependent ITP in a pediatric XLA patient, the use of thrombopoietin receptor agonists was considered [12]. If the ITP is steroid‐resistant or dependent, then the usual approach is to use thrombopoietin receptor agonists or rituximab to taper the dose of PSL as early as possible [4]. However, in ITP with XLA, there are no mature B cells, so rituximab is not expected to be effective, and the use of thrombopoietin receptor agonists is a reasonable strategy.

## 4 Conclusion

This report describes a rare case of ITP in an adult patient with XLA. Clinicians should keep ITP in mind when thrombocytopenia develops rapidly in a patient with agammaglobulinemia or XLA.

## Author Contributions

Takeaki Matsunaga, Ken Naganuma, and Yasushi Kubota were responsible for patient care and wrote the manuscript. Yasushi Kubota designed the study. Noriko Tanabe and Yoshiko Mori performed the genetic testing and counseling. Marino Nagata and Shuji Momose performed the pathological examination.

## Conflicts of Interest

The authors declare no conflicts of interest.

## Ethics Statement

The study was approved by the institutional review board of Saitama Medical University.

## Informed Consent

The patient provided informed consent.

## Clinical Trail Registration

Clinical trial registration is not needed for this submission.

## Data Availability

The data that support the findings of this study are available from the corresponding author upon reasonable request.
